# Changes of CD4+CD25+ Cells Ratio in Immune Organs from Chickens Challenged with Infectious Bursal Disease Virus Strains with Varying Virulences

**DOI:** 10.3390/v7031357

**Published:** 2015-03-20

**Authors:** Xiaoxue Yu, Lei Rui, Qiang Shao, Haiwen Liu, Yanan Lu, Yongchao Zhang, Zandong Li

**Affiliations:** State Key Laboratory of Agrobiotechnology, Department of Biochemistry and Molecular Biology, College of Biological Science, China Agricultural University, Beijing 100193, China; E-Mails: yuxiaoxue1990@163.com (X.Y.); ruilei@cau.edu.cn (L.R.); shaoqiang19880316@126.com (Q.S.); haiwen163@163.com (H.L.); nan0914@126.com (Y.L.); zhangyongchao09@163.com (Y.Z.)

**Keywords:** IBDV, CD4+CD25+ cells, migration, immune organs

## Abstract

In the current study, we investigate changes in CD4+CD25+ cells in chickens during infectious bursal disease virus (IBDV) infection. The percentage of CD4+CD25+ cells in lymph organs, e.g., the thymus, spleen, bursa of Fabricius and peripheral blood, during the first 1–5 days post infection (dpi) was assessed by flow cytometry. The data revealed a remarkable decrease in the percentage of CD4+CD25+ cells in the thymus from 1 to 5 dpi and in the spleen during early infection. An increase of the percentage of CD4+CD25+ cells among peripheral blood lymphocytes was observed during the first two days of IBDV infection. Additionally, CD4+CD25+ cells infiltrated the bursa along with CD4+ cells after IBDV infection. Quantitative reverse transcription polymerase chain reaction (qRT-PCR) was used to measure the mRNA levels of immune-related cytokines in IBDV-infected thymus and bursa of Fabricius tissues. The data revealed that IBDV caused a significant increase in interleukin (IL)-10 mRNA levels, with the Harbin-1 strain (vvIBDV) inducing higher IL-10 expression than the Ts strain. Taken together, our data suggest that chicken CD4+CD25+ cells may participate in IBDV pathogenicity by migrating from their sites of origin and storage, the thymus and spleen, to the virally targeted bursa of Fabricius during IBDV infection.

## 1. Introduction

Infectious bursal disease virus (IBDV) can lead to an acute, highly contagious immunosuppressive response in young chickens. Mammalian CD4+CD25+ regulatory T cells have been reported to play a critical immunosuppressive role in many diseases; however, there is limited information regarding the function of CD4+CD25+ cells in chickens during IBDV infection.

Infectious bursal disease (IBD), which is caused by IBDV, can lead to significant economic losses in the poultry industry [1]. IBDV belongs to the family *Birnaviridae* and consists of two segments, segments A (3.2 kb) and B (2.9 kb), which encode five proteins (VP1-VP5) [2,3]. IBDV can be differentiated into two serotypes (serotypes 1 and 2). Serotype 1 produces varying degrees of pathogenicity and mortality in chickens, whereas serotype 2 is avirulent in chickens [4,5,6]. Serotype 1 strains are classified as classical, intermediate, very or hyper-virulent. IBDV infection causes a lymphoid depletion of B cells and the destruction of bursal tissues, which are crucial to its immunosuppressive effect [7].

Regulatory T cells (Tregs) are a subset of T cells that specialize in immune suppression. The significance of Tregs in regulating the immune response was established in the 1990s [8,9,10]. CD4+CD25+FoxP3+ Tregs are a subset of Tregs that originate as a separate lineage of cells in the thymus [11].

Previous reports have demonstrated that various viruses may take advantage of host immune mechanisms associated with immunosuppressive functions to aid viral expansion and contribute to viral pathophysiology [12]. Viral infection can induce CD25 expression in lymphocytes [13] or directly activate CD4+CD25+ cells, potentially contributing to immune dysfunction [14]. Expanded Treg populations have been detected in many virus-related diseases, such as those caused by hepatitis C virus [15,16,17], hepatitis B virus [18,19], Epstein-Barr virus [20], and porcine reproductive and respiratory syndrome virus [21], as well as upon secondary infection with a virus [22]. Previous studies have postulated that suppressor T cells may be involved in the immunosuppression induced by IBDV [23,24].

Chicken CD4+CD25+ cells have been characterized as having similar suppressive and cytokine (IL-10 and TGF-β) production properties as mammalian regulatory T cells [25]. Our study aimed to investigate whether chicken CD4+CD25+ cells participate in IBDV-induced immunosuppression and pathophysiology. An anti-chicken CD25 monoclonal antibody (mAb) [26,27] was produced in mice and conjugated to a fluorescent R-phycoerythrin (RPE) tag. The specificity of the mAb against chicken CD25 was confirmed with flow cytometry [25] and Western blotting (WB). We also used two IBDV strains with different levels of virulence: a very virulent IBDV strain (Harbin-1) and a moderately virulent IBDV strain (Ts).

After challenge with IBDV, the percentages of CD4+CD25+ cells in different immune organs and in the peripheral blood were determined using flow cytometry, and the expression levels of immune-related cytokines were analyzed using quantitative reverse transcription polymerase chain reaction (qRT-PCR) assays.

## 2. Materials and Methods

### 2.1. Chickens and Viruses

Four-week-old specific pathogen-free (SPF) white leghorn chickens were purchased from Meria (Meria, Beijing, China) and housed in isolators; water and food were freely available. The animal welfare and experimental procedures adhered to the Institutional Guidelines of the Care and Use of Laboratory Animals at China Agricultural University (Beijing, China). All efforts were made to minimize suffering.

The Harbin-1 strain (vvIBDV) [28,29] was provided by the Harbin Veterinary Research Institute of the Chinese Academy of Agricultural Sciences. The Ts strain [29], a cell-adapted virus supplied by our laboratory, resulted in 0% mortality and was used as a moderately virulent reference strain. Virus propagation and the determination of the titers of both viral stocks were performed as previously described [29,30]. The Harbin-1 strain stock was 104.24 egg infective dose 50 (EID50) per 0.1 mL and was used as an inoculum following a 2-fold dilution. The tissue culture infectious dose 50 (TCID50) of the Ts strain was 104.7 per 0.1 mL and was used as an inoculum with no dilution. The SPF chickens were infected with 0.2 mL of inocula via eye drop application and nasal drip. We used PBS as the viral diluent and as a vehicle treatment in the control group.

### 2.2. Antibodies

We used an anti-CD16/32 antibody (eBioscience, San Diego, CA, USA) as an anti-Fc antibody, and a FITC-conjugated mouse anti-chicken CD4 antibody (Southern Biotech, Birmingham, AL USA) was purchased from SouthernBiotech, the isotype is mouse IgG1k.

The mouse anti-chicken CD25 antibody (anti-ch CD25) was produced as follows. The nucleotide sequence encoding the extracellular domain (aa 21–190) of chicken CD25 was amplified from chicken splenic cDNA via high-fidelity PCR using the following primers: forward, 5-CGGGGTACCGATAAATGCCCACGT-3, containing a KpnI site, and reverse, 5-CCCAAGCTTCTGCTTGTTTATAGG-3, containing a HindIII site. The PCR product was inserted into the pGEM®-T Easy Vector (Promega, Madison, WI, USA) according to the manufacturer’s instructions. The resulting ligation product was used to transform Top10 competent *E.coli* cells. Colonies were picked and sequenced using T7 and SP6 primers. One colony with the correct sequence and orientation was selected and grown. Plasmid DNA was extracted using the TIANprep mini plasmid kit (TIANGEN, Beijing, China) and digested with KpnI and HindIII. The digested PCR product was then inserted into the corresponding sites of the pET30a-GST expression vector. The pET30a-GST-CD25 vector was used to transform *E. coli* BL21 cells to express the extracellular domain of the chicken CD25 protein (aa 21–190) as a fusion protein carrying a glutathione S-transferase (GST) tag. The CD25-GST tag fusion protein was purified with a nickel column under denaturing conditions (Ni Sepharose 6 Fast Flow, GE Healthcare). The CD25-GST tag fusion protein was analyzed by sodium dodecyl sulfate polyacrylamide gel electrophoresis (SDS-PAGE) and Western blotting with an anti-GST tag mAb. Mouse anti-chicken CD25 was produced in collaboration with Beijing Protein Innovation Co., Ltd. (Beijing, China). The anti-chicken CD25 antibody isotype is IgG2a. The primary anti-chicken CD25 mAb was conjugated to RPE using an R-PE conjugation kit (Innova Biosciences, Cambridge, UK) according to the manufacturer’s instructions. The specificity of the mAb directed against chicken CD25 was studied by evaluating the Con A-induced CD25 upregulation in thymocytes and the thymus protein identified by the anti-chicken CD25 mAb.

To assess Con A-stimulated expression of CD25, single-cell suspensions of peripheral blood lymphocytes were isolated by density centrifugation over Hisstopaque (1.077 g/mL; Sigma-Aldrich, St. Louis, MI, USA). Lymphocytes were cultured in RPMI 1640 medium supplemented with 10% fetal bovine serum (FBS), 1% penicillin plus streptomycin, and 10 μg/mL Con A for 0 or 48 h. The cells were collected and washed with PBS. Then, the cells were incubated with anti-Fc antibody (anti-CD16/32, eBioscience, San Diego, CA, USA), washed with PBS, and incubated with 1 μL of fluorescein isothiocyanate (FITC)-conjugated mouse anti-chicken CD4 (Southern Biotech, Birmingham, AL, USA) and 1 μL of RPE-linked mouse anti-chicken CD25 in a 50-μL cell suspension (~106 cells) for 30 min on ice. The unbound Abs were removed by centrifugation. The percentages of CD4+CD25+ cells were analyzed with a flow cytometer (FACSCalibur, BD Bioscience, Franklin Lakes, NJ, USA).

### 2.3. Viral Infection and Collection of Samples

Four-week-old SPF chickens were randomly allotted into three groups and were housed in three isolators under the same conditions. Groups 1 (*n* = 30) and 2 (*n* = 25) were infected with the Harbin-1 strain and the Ts strain, respectively, via the eye and nose instillation routes. Each bird was inoculated with 0.2 mL of diluted virus. The chickens in group 3 (*n* = 25) were inoculated with 0.2 mL of PBS per bird to serve as controls. At 1–5 days post-infection (dpi), peripheral blood, bursal tissues, the thymus and the spleen (*n* = 5 per day) were collected separately from the infected and control groups. Peripheral blood lymphocytes were isolated using chicken peripheral blood lymphocyte-separation medium (TBD, Tianjin, China) according to the manufacturer’s instructions. The spleen, thymus and bursa of Fabricius were excised, and single-cell suspensions were separately prepared by crushing the organs. Lymphocytes were separated using a discontinuous density gradient of lymphocyte-separation medium (TBD).

### 2.4. Fluorescence-Activated Cell Sorting (FACS) Analysis of Lymphocytes

At 1, 3, and 5 dpi, CD4 and CD25 staining of the lymphocytes was performed. Following three washes with PBS, 2–3 × 105 cells were incubated with anti-chicken CD4-FITC or CD25-RPE mAb at 4 °C for 30 min for one-color staining. For two-color staining, 2–3 x 105 cells were incubated with anti-chicken CD4-FITC and CD25-RPE mAbs at 4 °C for 30 min. Subsequently, the cells were washed three times with PBS and were analyzed with a FACSCalibur (Becton Dickinson, Franklin Lakes, NJ, USA) using CellQuest (Becton Dickinson) and FlowJo (version 7.6.1) software. Viable lymphocytes were gated on the basis of forward and side scatter characteristics, and 10,000 or 20,000 events were analyzed for positive staining with FITC or PE.

### 2.5. Quantitative Real-Time PCR Analysis of Gene Expression

The qRT-PCR was performed in 15 μL of LightCycler@ 480 SYBR Green I Master Mix (Roche, Rotkreuz, Switzerland) with the LightCycler@ 480 Real-Time PCR System (Roche).

The individual primers used are shown in Table 1.

**Table 1:** Sequences of the primers used in qRT-PCR.

Gene	Direction	Sequence	GenBank Accession No.
GAPDH	Forward	GGTAGTGAAGGCTGCTGCTGAT	NM_204305.1
	Reverse	GGAGGAATGGCTGTCACCAT	
IL-10	Forward	GGCGACCTGGGCAACAT	NM_001004414.2
	Reverse	CCTTGATCTGCTTGATGGCTTT	
TGF-β	Forward	TGCGGCCAGATGAGCATATAG	M31154.1
	Reverse	GTGTCGGTGACATCGAAGGA	
Harbin-1 a	Forward	CACTCCCTGGTGGCGTTTA	EF517528.1
Ts b	Reverse	TGTCGTTGATGTTGGCTGTTG	AF076230.1
	Forward	ACCGGCACCGACAACCTTA	
	Reverse	CCCTGCCTGACCACCACTT	

a,b Primers from Haiwen Liu (1).

The cycling parameters were as follows: 95 °C for 10 min; 40 cycles of 95 °C for 15 s and 60 °C for 1 min; one cycle of 95 °C for 15 s, 60 °C for 15 s and 95 °C for 15 s; and one cycle of 40 °C for 30 s.

The expression levels of the genes of interest were calculated relative to the expression of the reference gene, glyceraldehyde 3-phosphate dehydrogenase (GAPDH). Increases or decreases relative to the untreated samples were expressed as fold changes, which were calculated using Microsoft® Excel 2010.

### 2.6. Data Analysis

Statistical analyses were performed with the Statistical Package for the Social Sciences (SPSS) version 20.1 software (SPSS Inc., Chicago, IL, USA). Independent-samples t-tests were used to test for significant differences between the IBDV-infected and control groups. Differences were regarded as significant at *P* ≤ 0.05.

## 3. Results

### 3.1. Production and Specific Detection of Mouse Anti-Chicken CD25 Antibody

**Figure 1:**
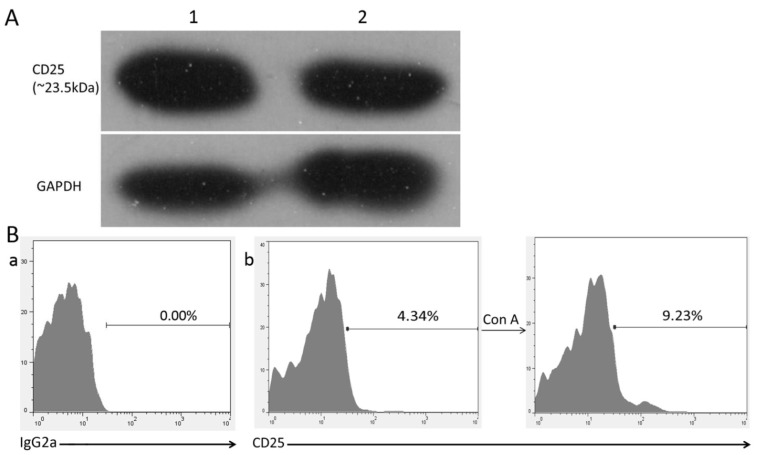
Specific detection by anti-chicken CD25 antibody. (**A**) Anti-chCD25 antibody recognized a specific chicken thymus protein band at approximately 23.5 kDa. Line 1: total thymus proteins from white leghorn chickens; line 2: total spleen proteins from white leghorn chickens; GAPDH (~36 kDa) was used as a control; (**B**) Staining of Con A-stimulated lymphocytes with anti-chCD25. (**a**): isotype control; (**b**): lymphocytes were treated with Con A for 0 or 48 h.

As the Western blotting result in Figure 1 shows, the anti-chicken CD25 mAb that we produced bound to the CD25 protein from chicken thymus and spleen, a protein band that was approximately 23.5 kDa (a specific band between 15 and 25 kDa), as expected based on the ExPASy data. The RPE-conjugated anti-chicken CD25 mAb recognized 4.2% (±0.12%) of the untreated peripheral blood lymphocytes and 9.1% (±1.25%) of the cells in the 48 h Con A-treated groups (*P* = 0.004) (*n* = 3; the experiment was performed in duplicate). This finding indicated that the anti-chicken CD25 antibody could be used for flow cytometric analyses.

### 3.2. Viral Load

**Figure 2:**
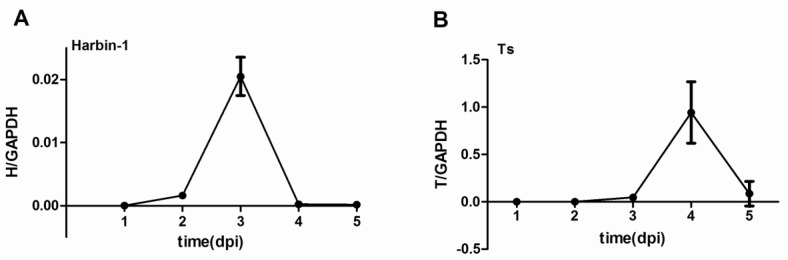
Vp2 mRNA levels in the bursa of Fabricius of SPF chickens infected with the Harbin-1 (**A**) or Ts strain (**B**) of IBDV.

Because the vp2 gene encodes one of the major structural proteins of IBDV, the level of vp2 mRNA is regarded as a measure of viral load. The expression levels of vp2 mRNA in the bursa of Fabricius of the IBDV-infected SPF chickens were quantitated via qRT-PCR assays. The data indicated that the vp2 mRNA levels increased dramatically after IBDV infection. The Harbin-1 viral load peaked at 3 dpi (Figure 2A), and the Ts viral load peaked at 4 dpi (Figure 2B). After reaching their peaks, the viral loads of both the Harbin-1 and Ts strains decreased.

### 3.3. CD4+CD25+ Cells Migrated Out of the Thymus and Spleen after IBDV Challenge and IL10 Expression Increased in the Thymus

**Figure 3:**
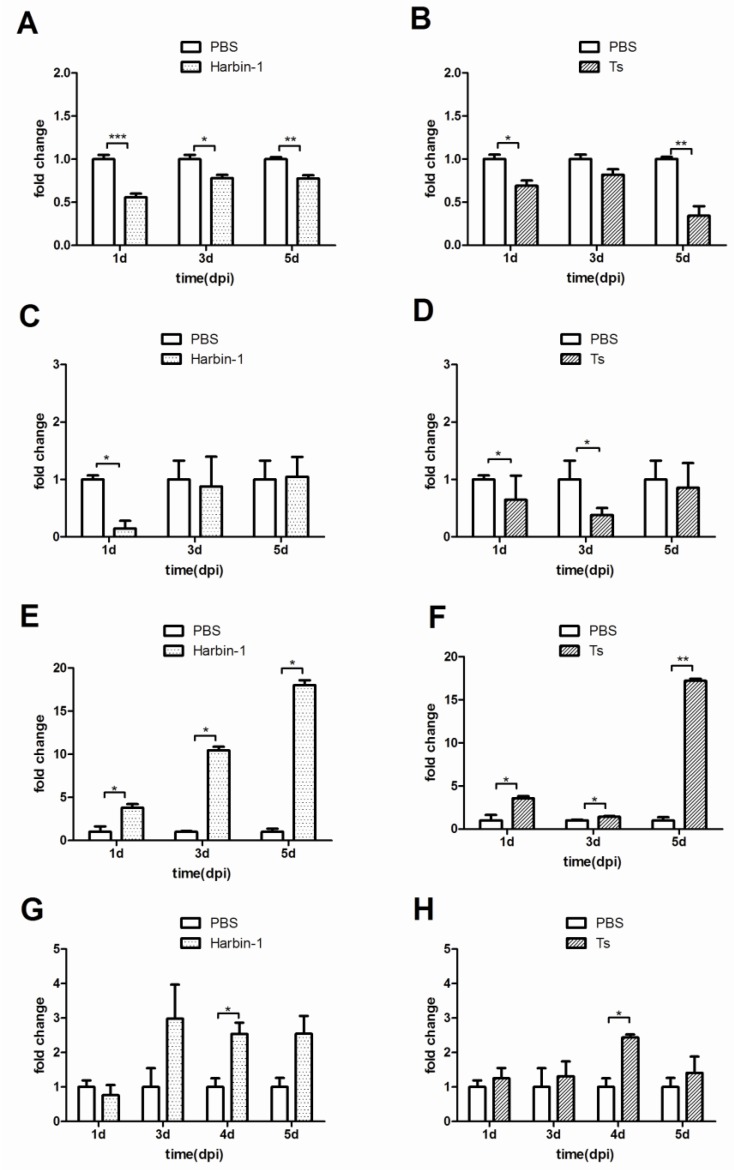
Changes in the CD4+CD25+ cell levels in the thymus and spleen and cytokine expression levels in the thymus of IBDV-infected SPF chickens. (**A**,**B**) Changes in the CD4+CD25+ cell level in the thymus of SPF chickens infected with the Harbin-1 or Ts strain; (**C**,**D**) Changes in CD4+CD25+ cell level in the spleen of SPF chickens infected with the Harbin-1 or Ts strain; (**E**,**F**) Changes in the IL-10 expression level in the thymus of SPF chickens infected with the Harbin-1 or Ts strain; (**G**,**H**) Changes in the TGF-β expression level in the thymus of SPF chickens infected with the Harbin-1 or Ts strain. Note: the y-axis indicates the fold change of the IBDV group/PBS group, and the x-axis shows the time after SPF chickens were infected with the Harbin-1 or Ts strain. Data represent mean ± SEM from 3–5 individuals. * *P* ≤ 0.05; ** *P* ≤ 0.01; *** *P* = 0.000.

Because CD4+CD25+ cells originate in the thymus, this organ has a significant proportion of CD4+CD25+ cells. After IBDV exposure (either Harbin-1 strain or Ts strain), CD4+CD25+ cells were detected from 1 dpi to 5 dpi. As depicted in Figure 3A,B, the proportion of CD4+CD25+ cells among all the CD4+ cells in the thymus decreased markedly after infection with either the Harbin-1 strain or the Ts strain. These results indicated that the CD4+CD25+ cells migrated out of the thymus upon IBDV infection.

Meanwhile, the proportion of CD4+CD25+ cells in the spleen exhibited a decrease on the first day of Harbin-1 infection and then returned to a normal level relative to the PBS group by 3 dpi and 5 dpi (Figure 3C). The proportion of CD4+CD25+ cells also exhibited an immediate decrease after Ts strain infection; however, in contrast to the Harbin-1 group, this proportion returned to a normal level by 5 dpi in the Ts group (Figure 3D). These data indicated that, after IBDV infection, CD4+CD25+ T cells in the spleen immediately migrated out. As the infection progressed, CD4+CD25+ cells were induced or migrated into the spleen, leading to a level similar to that of the PBS group.

We also assessed the *IL-10* and *TGF-β* mRNA expression levels in the thymus using qRT-PCR assays after an IBDV challenge. The data revealed that *IL-10* mRNA expression increased significantly at 1–5 dpi in the groups infected with either of the IBDV strains. In the Harbin-1 infection group, *IL-10* expression increased over time (Figure 3E). In the Ts infection group, the *IL-10* expression increased 17.2-fold at 5 dpi (Figure 3F). The *TGF-β* mRNA level also showed a two- to threefold increase in the Harbin-1 group and a one- to twofold increase in the Ts group; however, the difference was only significant at 4 dpi with the Harbin-1 strain (Figure 3G,H).

### 3.4. Fluctuation in the Proportion of CD4+CD25+ Cells in the Peripheral Blood Environment of IBDV-Infected Chicken

**Figure 4:**
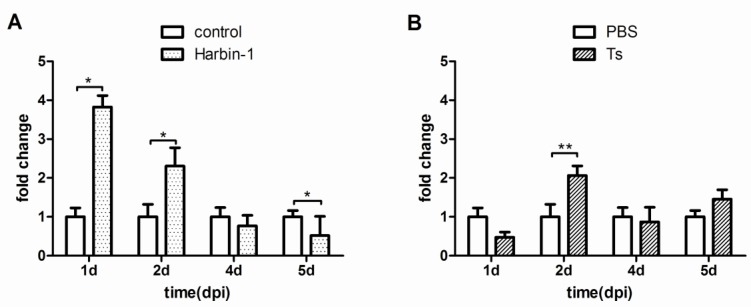
Changes in the level of CD4+CD25+ cells among peripheral blood lymphocytes of SPF chickens infected with the Harbin-1 or Ts strain. Note: the y-axis indicates the fold change of the IBDV group/PBS group, and the x-axis indicates the time after SPF chickens were infected with the Harbin-1 or Ts strain. Data represent mean ± SEM from 3–5 individuals. * *P* ≤ 0.05; ** *P* ≤ 0.01.

As illustrated in Figure 4, the proportion of CD4+CD25+ cells among peripheral blood lymphocytes increased during the first two days of either Harbin-1 infection or Ts infection. This result is consistent with the decrease of CD4+CD25+ cells in the thymus and spleen. Ultimately, normal levels were restored during the last two days of infection. This finding indicates that the CD4+CD25+ cells in the peripheral blood were either induced or migrated in and out as the infection continued.

### 3.5. CD4+CD25+ Cells Infiltrated the Bursa of Fabricius along with CD4+ Cells in Ts-Infected Chickens

**Figure 5:**
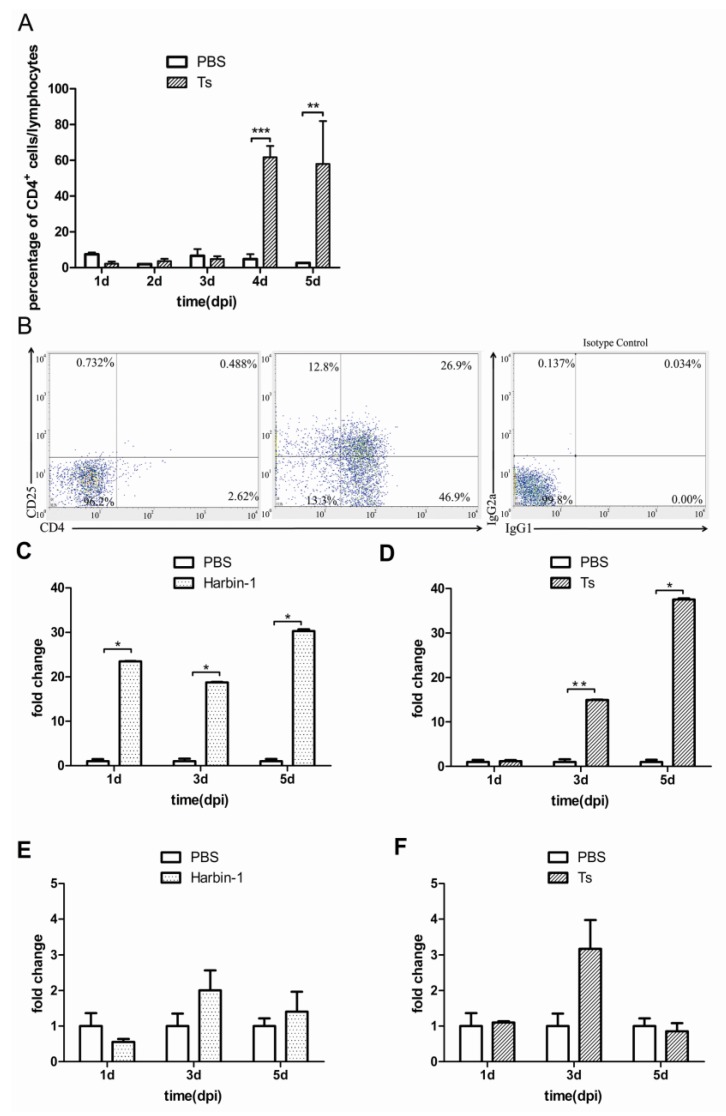
Changes in lymphocytes in the bursa of Fabricius from Ts-infected chickens and cytokines expression levels in IBDV-infected bursal tissues. (**A**) Changes in CD4+ cells in the bursa of Fabricius of SPF chickens infected with the Ts strain; (**B**) Ratio of CD4+CD25+ cells among the CD4+ cells in the bursa of Fabricius of SPF chickens infected with the Ts strain. Left: PBS control; middle: Ts-infected (4 dpi); right: isotype control; (**C**,**D**) Changes in *IL-10* expression in the bursa of Fabricius of SPF chickens infected with the Harbin-1 or Ts strain; (**E**,**F**) Changes of *TGF-*β expression in bursa of Fabricius of SPF chickens infected with the Harbin-1 or Ts strain. Note: the y-axis in Figure 5A indicates the ratio of CD4+ cells/lymphocytes; the y-axis in Figure 5C–F indicates the fold change of the IBDV group/PBS group, and the x-axis means the time after Harbin-1 or Ts strain-infected SPF chickens. Data represent mean ±SEM from 3–5 individuals. * *P* ≤ 0.05; ** *P* ≤ 0.01.

The bursa of Fabricius is the main target organ of IBDV. Harbin-1 strain (vvIBDV) infection caused serious pathological bursal lesions, and few lymphocytes could be collected. Therefore, we collected and analyzed the CD4+CD25+ cells in the bursa of Fabricius from Ts strain-infected chickens. As shown in Figure 5, CD4+ T cells represented less than 10% of the bursal lymphocytes in the PBS group, and CD4+CD25+ cells were almost undetectable in the PBS group. Following IBDV infection, CD4+CD25+ cells could be detected. As Figure 5A,B indicate, CD4+ T cells increased after the Ts strain infection and constituted more than 60% of the bursal lymphocytes at 4 dpi and 5 dpi, and CD4+CD25+ cells represented a large proportion (44.3% ± 7.4%) of the bursa-infiltrating CD4+ cells. This finding indicates that CD4+CD25+ cells infiltrated the bursa of Fabricius along with CD4+ T cells after IBDV infection.

A large increase in the expression of IL-10 mRNA was observed from 1 dpi to 5 dpi after Harbin-1 infection (Figure 5C). In contrast, the expression of IL-10 increased beginning 3 dpi after Ts infection (Figure 5D). The TGF-β mRNA levels in the bursa of Fabricius had a one- to twofold increase in the Harbin-1 infection group (Figure 5E) and an approximately three-fold increase in the Ts infection group (Figure 5F).

## 4. Discussion

IBD, which is caused by IBDV, is an acute, highly contagious immunosuppressive disease of young chickens. IBDV can induce both humoral and cellular immunosuppression [31,32]. The destruction of immunoglobulin-producing cells by IBDV leads to the humoral immunosuppression [32]. The principal organ for the replication of IBDV is the bursa of Fabricius [33]. As one of the main structural proteins, VP2 is very important for the pathogenicity of IBDV [34,35]. Therefore, in this study, we regarded the mRNA level of *vp2* as an indicator of the IBDV viral load. The IBDV *vp2* mRNA expression levels we detected were correlated to the amount of viral exposure. The Harbin-1 strain viral load peaked at 3 dpi, and the Ts viral load peaked at 4 dpi (Figure 2) before subsiding, in accordance with the trends noted in our previous study [1]. The viral load of the Harbin-1 strain following a large inoculum was considerably lower the viral load following a lesser inoculum in a previous study in our laboratory [1] (Figure 2). This finding may have been due to the depletion of IgM-producing B lymphocytes. Our data indicated that a lower viral load of vvIBDV (Harbin-1 strain) could still induce a more severe immune response compared with the Ts strain.

In mammals, Tregs play indispensable roles in maintaining immunological unresponsiveness to self-antigens and in suppressing excessive immune responses that would be deleterious to the host. Treg markers include CD4, CD25 and FOXP3. Because *foxp3* localizes to the nucleus, CD4 and CD25 are regarded as surface markers of Tregs. No *foxp3* gene homolog has been identified in chickens to date [36], but chicken CD4+CD25+ cells have been characterized as having immunosuppressive and cytokine-secreting functions similar to those in mammals [25]. We generated a mouse anti-chicken CD25 antibody. The antibody specificity was confirmed via Western blotting and flow cytometry.

In the absence of virus-specific antibodies, cell-mediated immunity (CMI) is generally adequate in protecting birds against virulent IBDV. This finding demonstrates that CMI plays a very important role in the pathogenesis of IBDV [37]. Many studies of the immune system have suggested the presence of immunoregulatory cells in the chicken thymus [38]. In our study, the number of CD4+CD25+ cells in the thymus decreased immediately following IBDV infection (either Harbin-1 or Ts strain). We believe that the viral infection led those regulatory CD4+CD25+ cells to migrate to the periphery away from their natural origin to effect their suppressive roles. Consistent with these results for CD4+CD25+ cells in the thymus, increased numbers of CD4+CD25+ cells were detected within the peripheral blood beginning at 2 dpi. *IL-10* and *TGF-*â expression increased in the thymus with either Harbin-1 or Ts strain infection. While vvIBDV, the Harbin-1 strain, induced *IL-10* expression more potently than the Ts strain. The precise immunosuppressive mechanism of T regulatory cells remains elusive; however, there is increasing evidence that Tregs manifest their function through a myriad of mechanisms, including the secretion of immunosuppressive soluble factors, such as IL-10 and TGF-â; cell contact-mediated regulation via the high affinity TCR and other costimulatory molecules, such as CTLA-4, and GITR; and cytolytic activity. In our study, the number of CD4+CD25+ cells in the thymus decreased, whereas IL10 and TGF-â expression increased, after IBDV infection. Thus, we hypothesize that cytokines are produced by CD4+CD25+ cells that remained or were induced in the thymus.

The spleen, as a secondary immune organ, plays an important role in responding to antigens. Splenic CD4+CD25+ T cells also decreased immediately upon IBDV infection and then returned to a normal level relative to the PBS group. However, whether the increased proportion of CD4+CD25+ T cells was due to induction or migration could not be precisely determined. A previous study has found that the splenocytes from IBDV-infected chickens at 7 dpi had no detectable virus-specific proliferation [39]. In combination with our data, these results indicate to us that the spleen may act largely as peripheral lymphocyte storage during IBDV infection; while we need more experimental evidence to confirm this. In peripheral regions, such as the peripheral blood environment, CD4+CD25+ T cell levels displayed an interesting fluctuation. CD4+CD25+ cells in the peripheral blood were decreased by the first day of IBDV infection and subsequently increased to a normal level.

Previous studies have shown that T cells infiltrate into the bursa (the IBDV target site) following IBDV infection [39,40], which suggests that T cells may play a role in the pathogenesis of IBDV infection; however, the specific T cell subpopulations have never been fully analyzed. In the bursa of Fabricius of the PBS-treated control chickens, few T lymphocytes were detected (Figure 5A). In contrast, a large proportion of CD4+CD25+ cells among the infiltrated CD4+ T cells were detected in the bursa of Fabricius of the IBDV-infected chickens upon infection with the Ts strain. However, vvIBDV infection (Harbin-1 strain) caused serious pathological bursal lesions, and few lymphocytes could be collected to analyze the proportion of CD4+CD25+ cells. The *IL-10* mRNA expression levels were significantly increased at the bursa site, in agreement with previous results from our laboratory [1]. The Harbin-1 strain induces *IL-10* expression more potently than the Ts strain in the bursa of Fabricius, the main target organ of IBDV.

In summary, chicken CD4+CD25+ cells, which are considered similar to Tregs in chickens, exhibited interesting changes following IBDV infection. We concluded that the CD4+CD25+ cells in the thymus and spleen migrated out to the periphery following IBDV infection to effect their immunosuppressive role during the early stages of infection. However, the number of CD4+CD25+ cells in the spleen quickly returned to normal levels (although it is unclear whether these CD4+CD25+ cells were induced or migrated from the thymus). The bursa of Fabricius (the main site of viral replication) showed evidence of infiltration by CD4+CD25+ cells along with CD4+ cells. These data demonstrate that CD4+CD25+ cells participate in IBDV infection. However, the exact ratio of induced to migratory CD4+CD25+ cells and the mechanism by which chicken CD4+CD25+ cells carry out their role should be further investigated in future studies. Understanding the mechanisms by which CD4+CD25+ cells exert their influence during IBDV infection can give us implications for the development of therapeutic strategies for many other diseases including cancer, diabetes, and immune mediated diseases.
